# Effect of climatic variation on the morphological characteristics of 37‐year‐old balsam fir provenances planted in a common garden in New Brunswick, Canada

**DOI:** 10.1002/ece3.3852

**Published:** 2018-02-19

**Authors:** Matthew E. Akalusi, Charles P.‐A. Bourque

**Affiliations:** ^1^ Faculty of Forestry and Environmental Management University of New Brunswick Fredericton NB Canada

**Keywords:** climate model, climate normals, intraspecific variation, latitude, plant–climate interactions, population, species range

## Abstract

The extent of the effect of projected changes in climate on trees remains unclear. This study investigated the effect of climatic variation on morphological traits of balsam fir [*Abies balsamea* (L.) Mill.] provenances sourced from locations spanning latitudes from 44° to 51°N and longitudes from 53° to 102°W across North America, growing in a common garden in eastern Canada. Lower latitude provenances performed significantly better than higher latitude provenances (*p* < .05) with regard to diameter at breast height (DBH), height (H), and crown width (CW), a distinction indicative of genotypic control of these traits. There was, however, no significant difference among provenances in terms of survival (*p *>* *.05), an indication of a resource allocation strategy directed at survival relative to productivity in higher latitude provenances as seen in their lower DBH, H, and CW compared to the lower latitude provenances. Temperature had a stronger relationship with DBH, H, and CW than precipitation, a reflection of adaptation to local conditions in populations of the species along latitudinal gradients. Both climatic variables had some effect on tree survival. These results suggest that the response of balsam fir to climatic variation will likely not be uniform in the species, but differ based on genetic characteristics between populations located in the northern and southern parts of the species’ range. Population differences in response to climatic variation may be evident earlier in growth traits, compared to survival in balsam fir. The findings of this study will facilitate modeling in the species that is reflective of genetic variation in response to climatic conditions, and guide provenance selection for utilization in terms of productivity or resilience as well as breeding programs directed at obtaining species that possibly combine both traits.

## 1 INTRODUCTION

Climate is a major environmental factor that controls the distribution and growth of plant species (Woodward, 1987). Species occupying large ranges which also span more than one climate zone usually show large intraspecific variation in physiology, morphology, and growth rate (Abrams, 1994; Palmroth, Berninger & Nikinmaa, 1999; Aspelmeier & Leuschner, 2004; Donselman & Flint, 1982; Geber & Dawson, 1993; Schuler 1994; Marchin, Sage, & Ward, 2008).Climate models predict a rise in the mean annual temperature of the Northern Hemisphere and modified patterns of precipitation (Andalo, Beaulieua, & Bousquet, 2005). North America is projected to warm by between 1 and 3°C this century, with the greatest warming expected to occur at high latitudes in winter and the southwest of the United States (US) in summer. Annual precipitation is projected to increase across the North American continent, except in the southwest of the United States where a decrease is anticipated, and parts of southern Canada where precipitation declines are expected to occur in summer and fall (Intergovernmental Panel on Climate Change, 2007; Lemmen, Warren, Lacroix, & Bush, 2008; Warren & Lemmen, 2014). It is projected that climate change will have a tremendous effect on forest ecosystems and tree growth. Iverson and Prasad (1998) based on scenario analysis, involving several conifer and broad‐leaved species in the United States, show potential shifts arising from climate change, which may result in species range transformations. Briffa Schweingruber, Jones, Osborn, Harris, et al. (1998); Briffa, Schweingruber, Jones, Osborn, Shiyatov, et al. (1998) in Northern Hemisphere tree ring studies showed increasing divergence between ring width and maximum latewood density and temperature variation over decadal scales. The extent of these anticipated changes, however, remains unclear (Saxe, Cannell, Johnsen, Ryan, & Vourlitis, 2001; Solberg, Hofgaard, & Hytteborn, 2002;  Wilson & Elling, 2004;  Büntgen et al., 2006; Savva, Bergeron, Denneler, Koubaa, & Tremblay, 2008).

Provenance trials, originally established in many countries in the last century for the selection of superior commercial genotypes, have emerged serendipitously as in situ laboratories for the study of tree response to climate change (Matyas, 1994; O'Neill & Nigh, 2011). Provenance trials involve the transfer of seeds from different parts of a species range to a similar environment, and as a result, simulate spatially the complex atmospheric variations likely to occur over the next few decades (Anderson, Panetta, & Mitchell‐Olds, 2012; Montesinos‐Navarro, Wig, Xavier Pico, & Tonsor, 2011), making them well‐suited for studying tree response to climate change (Schmidtling, 1994; Carter, 1996; Matyas, 1996, 1999; Persson, 1998;  Rehfeldt, Tchebakova, & Barnhardt, 1999; Andalo et al., 2005; Savva et al., 2008). Plant species comprising populations genetically attuned to different climates will experience short‐ and long‐term impacts on their growth and survival when such populations are moved from their climate of origin to a different climate (Rehfeldt, 2004). Short‐term impacts are controlled by the ability of species to make phenotypic adjustments to environmental change, while the long‐term response of forest trees to climate is achieved through processes such as selection, migration, and random genetic drift, which result in modification of gene pools (Rweyongeza, Yang, Dhir, Barnhardt, & Hansen, 2007). Using provenance trials, species or population responses across an environmental gradient can be characterized by relating the provenance's performance to climatic conditions at its source area. Effects of climatic change on future performance of species or populations can be predicted by modeling these response patterns, facilitating forest management strategies that can be based on knowledge of the adaptive capabilities of these species (Cherry & Parker, 2003; Thomson & Parker, 2008).

Balsam fir is a shade‐tolerant tree with a range that spans Canada (from Newfoundland to Alberta) and the United States (a substantial part of the northeast, extending to Minnesota and Virginia). It is used in pulp production, light frame construction, paneling, and the manufacture of medicines, and is popular as a Christmas tree (Frank, 1990). Several studies have modeled the response of conifers to climatic change using provenance trials based on jack pine (*Pinus banksiana*, Rweyongeza, Dhir, Barnhardt, Hansen, & Yang, 2007; Savva, Denneler, Koubaa, Tremblay, & Tjoelker, 2007; 2008; Tjoelker, Oleksyn, Reich, & Zytkowiak, 2008), white spruce (*Picea glauca*, Andalo et al., 2005; Rweyongeza, Yang, et al., 2007), black spruce, *Picea mariana*, Wei, Han, Dhir, & Yeh, 2004; Thomson, Riddell, & Parker, 2009), and Scots pine (*Pinus sylvestris*, Reich, Oleksyn, & Tjoelker, 1996; Persson, 1998; Rehfeldt et al., 2002). Such studies based on balsam fir are uncommon (Carter, 1996). The objectives of this study were to (i) determine the effect of climatic variation on morphological traits of balsam fir and (ii) develop climate response models for this species.

## 2 MATERIALS AND METHOD

### 2.1 Test site and Provenances

This study is based on data from a provenance trial established in 1961, made up of twelve balsam fir [*Abies balsamia* (L.) Mill.] seed sources planted at Salmon River Balsam Fir Provenance Research Plantation in northern New Brunswick, Canada (47° 7′N and 67° 32′W; MacGillivray, 1963). The test site is located in the Atlantic Maritime Ecozone (Ecological Stratification Working Group 1996), with the following climatic conditions for the period 1971‐2000 (i) mean annual temperature of 3.5°C; (ii) mean summer temperature of 16.8°C; (iii) mean winter temperature of −11.1°C; and (iv) total precipitation of 1134.4 mm (Environment Canada 2016). The provenances were sourced from locations spanning latitudes from 44° to 51°N and longitudes from 53° to 102°W across North America (Table 1).

**Table 1:** Provenance sources and provenance test site, with their coordinate position, key climatic variables for the period 1971‐2000 (mean annual temperature (MAT), mean winter temperature (MWT), mean summer temperature (MST), all in °C; total annual precipitation (TPPT), in mm), and Ecozone/Ecoregion location (refer to MacGillivray, 1963; Bailey, 1995; Ecological Stratification Working Group 1996; and Environment Canada 2016)

Provenance	Source	Latitude	Longitude	MAT (°C)	MWT (°C)	MST (°C)	TPPT (mm)	Ecozone/Ecoregion
MS‐130	Duck Mountain, Saskatchewan (SK)	51° 50ʹN	102°W	1.6	−15.5	11.1	450.9	Prairies
MS‐133	Roddickton, Newfoundland (NF)	50° 55ʹN	56°W	2.1	−9.2	13.2	975.3	Boreal Shield
MS‐131	Airplane Bay, Manitoba (MB)	50° 40ʹN	100°W	1.1	−16.1	16.4	457.1	Prairies
MS‐126	Hawke's Bay, Newfoundland (NF)	50° 37ʹN	57° 15ʹW	2.4	−8.4	12.7	1145.2	Boreal Shield
MS‐127	Bonne Bay, Newfoundland (NF)	49° 25ʹN	57° 44ʹW	4.1	−6.5	14.7	1620.7	Boreal Shield
MS‐123	Sandy Brook, Newfoundland (NF)	48° 44ʹN	56° 04ʹW	3.2	−7.8	14.1	1082.8	Boreal Shield
MS‐2	Green River Watershed, New Brunswick (NB)	47° 46ʹN	68° 15ʹW	3.2	−11.2	16.5	1091.5	Atlantic Maritime
MS‐125	Salmonier, Newfoundland (NF)	47° 17ʹN	53° 20ʹW	4.9	−3.7	13.3	1392.1	Boreal Shield
Test Site	Salmon River Plantation, New Brunswick (NB)	47° 07ʹN	67° 32ʹW	3.5	−11.1	16.8	1134.4	Atlantic Maritime
MS‐124	Valcartier Forest Station, Quebec (QC)	46° 55ʹN	71° 32ʹW	4.5	−10.5	18.2	1139.8	Atlantic Maritime
MS‐118	Acadia Research Forest, New Brunswick (NB)	45° 59ʹN	66° 21ʹW	5.0	−8.4	17.4	1202.7	Atlantic Maritime
MS‐117	Oromocto, New Brunswick (NB)	45° 52ʹN	66° 24ʹW	5.4	−7.9	17.9	1152.1	Atlantic Maritime
MS‐303	Adirondack Mountains, New York (NY)	44° 42ʹN	74°W	6.2	−6.8	18.4	1102.0	Warm Continental

### 2.2 Experimental design and data collection

The layout is a block design with three replications. Each block is made up of twelve 0.04 ha plots in which 100 trees were planted at a spacing of 1.8 m × 1.8 m in 10 rows of 10 trees. A total number of 3,600 trees were planted covering 0.48 ha.

In 1998, survival (%) per provenance plot was calculated, as well as 37‐year diameter at breast height (DBH, cm), height (H, m), and crown width (CW, cm) of all sampled trees. Mean values for each variable were calculated per provenance plot per block, and averaged across blocks for provenance means.

### 2.3 Analysis of Variance (ANOVA)

Analysis of variance (ANOVA) was used for DBH, H, CW, and survival using the General Linear Model option in SPSS Statistical software (ver. 24, IBM Corp., New York, USA), with provenance and block as fixed effects. Levene's test for homogeneity of sample variances was conducted. Analysis of variance was performed to determine the level of significance of the effect of provenance and blocking on the four tree variables. If the analysis of variance detected significant differences, the least significant difference (LSD) post hoc test was subsequently used to separate effect means.

### 2.4 Regression models

Statistical models were developed for 37‐year DBH, H, CW, and survival of balsam fir. Development of each model was based on the methods of Matyas and Yeatman (1992), Rehfeldt et al. (2002), Rweyongeza, Yang, et al. (2007), and Thomson and Parker (2008). Climatic variables for the period 1971‐2000 (described as climate normals) were obtained from weather station data (Environment Canada 2016; United States National Oceanic and Atmospheric Administration 2016) and used to relate provenance growth to climate at provenance origin. A total of 53 climatic variables were examined, including annual and seasonal (winter, spring, and summer) temperature‐based (18 variables, in total) and precipitation‐based variables (10); heat accumulation indices; growing degree days above 5 and 10°C (2); annual and seasonal moisture indices (8); durations above temperature (5) and precipitation thresholds (9); and a continentality index based on the difference between the warmest and coldest months in a year. The annual and seasonal moisture indices were calculated from the ratio of growing degree days >5 and 10°C to precipitation over the course of a year or in respective seasons. Values obtained were indicative of temperature levels and their effect on moisture availability, with higher values representative of areas with warm or hot summers with a potential for moisture deficits, and lower values representative of areas with cooler conditions (Rweyongeza, Dhir, et al., 2007). Based on prior visual review of scatter plots, linear regression (equation (1)) was used in assessing the relationship between CW and the climatic variables, whereas linear and quadratic regressions (equation (2)) were used in assessing the relationship between DBH, H, and survival, and the same suite of variables.

Provenance response to climate was assessed using regressions of each trait on a climatic variable at the provenance source area:

(1) $$Y=\beta_{0}+\beta_{1}X+\varepsilon$$

(2) $$Y=\beta_{0}+\beta_{1}X+\beta_{2}X^{2}+\varepsilon$$

Dependent variable *Y* in equations (1) and (2) is the provenance trait; independent variable *X* is the explanatory climatic variable for the provenance, β_0_, β_1_, and β_2_ are regression coefficients to be estimated, and ε is the error term for the provenance source. Based on the results of linear and quadratic regressions, climatic variables suitable for model development for balsam fir were retained, based on *r* ^2^‐values ≥.40 and *p*‐values <.05.

## 3 RESULTS

### 3.1 Provenance

The results show that provenance of 37‐year‐old balsam fir had a significant effect on DBH, H, and CW (*p *<* *.05), but no significant effect on survival (*p *>* *.05) on survival. Provenances sourced from Oromocto and NY were the best performing compared to other provenances (*p *<* *.05) in terms of DBH and H. Provenances sourced from Oromoctoand QC were the best performing, compared to other provenances in terms of CW (*p* < .05). The best performing provenances were all sourced from locations south of the study site Table 2. Generally, provenances sourced from locations south of the study site (hereafter, lower latitude provenances), and as a result moved to a cooler location, performed better than provenances sourced from locations north of the study site (hereafter, higher latitude provenances), moved to a warmer location.

**Table 2:** Mean DBH (cm), H (m), CW (cm) and survival (%) of 37‐year‐old balsam fir provenances growing in a common garden in northern New Brunswick; ±standard deviations are in parenthesis

Provenance	Mean DBH (cm)	Mean H (m)	Mean CW (cm)	Mean survival (%)
MS‐2	13.19 (±0.86)	12.09 (±0.43)	258.40 (±25.90)	76.33 (±5.86)
MS‐117	14.78 (±0.54)	12.93 (±0.05)	288.51 (±29.46)	80.33 (±5.69)
MS‐118	13.01 (±0.57)	10.91 (±0.51)	247.74 (±11.87)	76.00 (±3.46)
MS‐123	12.73 (±1.42)	10.60 (±0.78)	236.67 (±18.08)	77.33 (±3.06)
MS‐124	13.29 (±0.69)	12.29 (±0.68)	283.37 (±17.80)	78.67 (±7.02)
MS 125	11.39 (±0.64)	9.77 (±0.53)	278.47 (±5.58)	72.67 (±4.04)
MS‐126	11.13 (±0.43)	10.01 (±0.35)	246.69 (±4.07)	77.67 (±3.79)
MS‐127	12.09 (±0.75)	10.58 (±0.39)	260.37 (±26.45)	69.33 (±2.31)
MS‐130	13.15 (±0.82)	11.83 (±0.43)	216.12 (±25.73)	77.00 (±2.65)
MS‐131	11.72 (±0.48)	10.73 (±0.51)	234.34 (±7.81)	69.00 (±8.54)
MS‐133	12.90 (±0.40)	11.02 (±0.53)	269.67 (±20.74)	75.00 (±17.09)
MS‐303	14.49 (±0.44)	13.08 (±0.70)	262.56 (±31.06)	80.00 (±1.73)

### 3.2 Climatic variables

**Figure 1:**
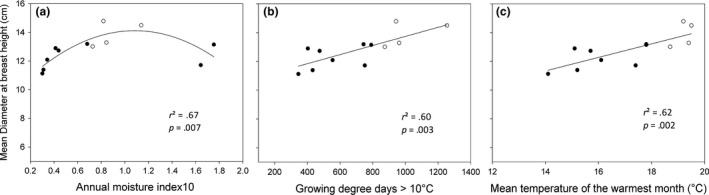
Diameter at breast height (cm) of 37‐year‐old balsam fir provenances in northern New Brunswick in relation to (a) annual moisture index based on GDD10, (b) mean temperature of the warmest month, and (c) GDD10 (refer to Table 4 for variable definition) at the point of origin. Lower latitude provenances are denoted by open circles, whereas higher latitude provenances are denoted by closed circles

**Figure 2:**
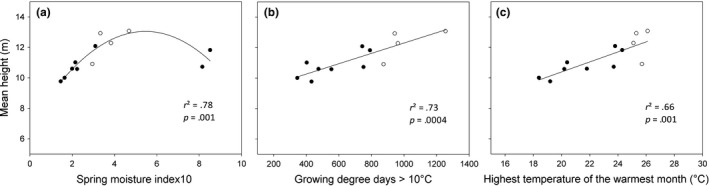
Tree height (m) of 37‐year‐old balsam fir provenances in northern New Brunswick in relation to (a) spring moisture index based on GDD10, (b) GDD10, and (c) the highest temperature of the warmest month at the point of origin. Lower latitude provenances are denoted by open circles, whereas higher latitude provenances are denoted by closed circles

**Figure 3:**
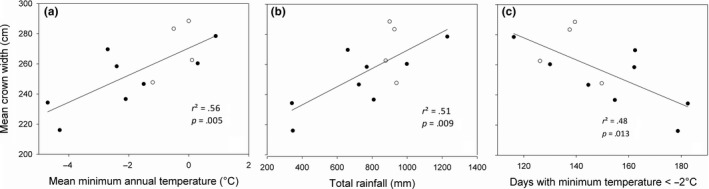
Crown width (cm) of 37‐year‐old balsam fir provenances in northern New Brunswick in relation to (a) the mean minimum annual temperature, (b) total rainfall, and (c) the days with minimum temperature <−2°C at the point of origin. Lower latitude provenances are denoted by open circles, whereas higher latitude provenances are denoted by closed circles

**Figure 4:**
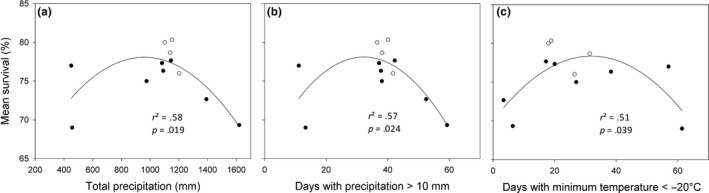
Mean survival (%) of 37‐year‐old balsam fir provenances in northern New Brunswick in relation to (a) total precipitation, (b) the number of days with precipitation >10 mm, and (c) the number of days with temperature <−20°C at the point of origin. Lower latitude provenances are denoted by open circles, whereas higher latitude provenances are denoted by closed circles

Annual moisture index based on GDD10 had the strongest influence on DBH (*r* ^2^ = .67) in a nonlinear relationship (for variable definition, refer to Table 4; consult Figure 1a for relationship). Spring moisture index based on GDD10 had the strongest influence on H (*r* ^2^ = .78), also in a nonlinear relationship (Figure 2a). Maximum DBH and H occurred at moderate annual and spring moisture indices based on GDD10, respectively, with lower latitude provenances sourced from Oromocto, NB, and Valcartier, QC, in the Atlantic Maritime Ecozone of Canada, and Adirondack, NY, in the Warm Continental Ecoregion of the United States, while the lowest values were obtained with higher latitude provenances sourced from Salmonier and Hawke's Bay, NF, in the Boreal Shield Ecozone of Canada. Mean minimum annual temperature had the strongest influence on CW (*r* ^2^ = .56), with values increasing linearly (Figure 3a). Crown width values increased with decreasing mean minimum annual temperature. Maximum CW was obtained with the lower latitude provenances sourced from Oromocto, NB, and Valcartier, QC, whereas the lowest CW values were obtained in the higher latitude provenances sourced from Airplane Bay, MB, and Duck Mountain, SK. The ecozone of the NB and QC provenances is as indicated earlier, and the MB and SK provenances were sourced from the Prairie Ecozone of Canada. Total precipitation had the strongest influence on survival (*r* ^2^ = .58) in a nonlinear relationship (Figure 4a). Maximum survival occurred at moderate TPPT with lower latitude provenances sourced from Oromocto, NB, and Valcartier, QC, in the Atlantic Maritime Ecozone of Canada, and Adirondack, NY, in the Warm Continental Ecoregion of the United States, whereas the lowest values were obtained with higher latitude provenances sourced from Bonne Bay, NF, and Airplane Bay, MB, in the Boreal Shield Ecozone of Canada (Table 3).

**Table 3:** Coefficients (β_0_, β_1_, β_2_, *r* ^2^) and *p*‐value from regressions of mean DBH (cm), H (m), CW (cm), and survival (%) of 37‐year‐old balsam fir in northern New Brunswick, in relation to climatic variables at the point of origin. Climatic variable abbreviations appear in Table 4

Trait	Climatic variable	β_0_	β_1_	β_2_	*r* ^2^	*p*
Mean DBH (cm)	AMI10	9.351	9.418	−4.410	.67	.007
	MTWM	4.752	0.470		.62	.002
	GDD10	10.582	0.003		.60	.003
	SpMAX	10.294	0.331		.56	.005
	MMAX	9.042	0.432		.53	.007
Mean H (m)	SpMI10	7.063	2.160	−0.197	.78	.001
	GDD10	8.905	0.003		.73	.0004
	HTWM	3.800	0.329		.66	.001
	LTWM	4.596	0.587		.51	.009
	SpMAX	8.766	0.335		.59	.003
	SMI10	9.366	0.797		.50	.010
Mean CW (cm)	MMIN	270.473	8.993		.56	.005
	TR	209.047	0.060		.51	.009
	DMIN ‐2	365.847	−0.733		.48	.013
Mean survival (%)	TPPT	59.335	0.039	−0.00002	.58	.020
	DPPT10	65.697	0.770	−0.012	.56	.024
	DMIN ‐20	70.010	0.521	−0.008	.51	.039
	WPPT	71.094	0.048	−0.0001	.50	.046

**Table 4:** Climatic variables and abbreviations

Parameter	Abbreviation
Mean maximum annual temperature	MMAX
Mean minimum annual temperature	MMIN
Mean maximum spring temperature	SpMAX
Highest temperature of the warmest month	HTWM
Lowest temperature of the warmest month	LTWM
Mean temperature of the warmest month	MTWM
Days with minimum temperature <−20°C	DMIN ‐20
Days with minimum temperature <−2°C	DMIN ‐2
Total precipitation	TPPT
Winter precipitation	WPPT
Total rainfall	TR
Days with precipitation above 10 mm	DPPT10
Growing degree days >10°C	GDD10
Annual moisture index based on GDD10	AMI10
Spring moisture index based on GDD10	SpMI10
Summer moisture index based on GDD10	SMI10

Also, the mean temperature of the warmest month, maximum spring temperature, maximum annual temperature, and GDD10 correlated fairly well with DBH. Diameter at breast height increased linearly with the mean temperature of the warmest month and GDD10 (Figure 1b,c). The spring and summer moisture indices based on GDD10, GDD10, the highest and lowest temperatures of the warmest month, and mean maximum spring temperature had good relationships with H. Tree height increased linearly with GDD10 and the highest temperature of the warmest month (Figure 2b,c). The mean minimum annual temperature, total rainfall, and days with minimum temperature <−2°C had good relationships with CW. Crown width increased with total rainfall, but decreased linearly in relation to days with minimum temperature < −2°C (Figure 3b,c). The number of days with precipitation above 10 mm, days with minimum temperature < −20°C, and winter precipitation had good relationships with survival, which were all nonlinear. Maximum survival was obtained in relation to moderate number of days with precipitation >10 mm and temperature below −20°C (Figure 4b,c).

## 4 DISCUSSION

The latitudinal distinction between lower and higher latitude provenances with regard to DBH, H, and CW is indicative of genotypic control of these traits arising from adaptations developed at their respective seed sources. Growing season length between spring bud‐burst and autumn leaf‐fall is well associated with latitudinal gradients (Elmore, Guinn, Minsley, & Richardson, 2012; Lechowicz, 1984; Thiel et al., 2012). Lower latitude provenances originate from warm more southerly environments with a longer growing season, characterized by earlier spring bud‐burst and later bud‐setting in fall (Davis & Shaw, 2001; Giertych & Oleksyn, 1981, 1992; Johnsen, Seiler, & Major, 1996; O'Neill & Yanchuk, 2005; Oleksyn, Zytkowiak, Karolewski, Reich, & Tjoelker, 2000; Repo, Zhang, Ryyppo, Rikala, & Vuorinen, 2000; Savva et al., 2007; Shutyaev & Giertych, 1997), whereas higher latitude provenances originate from cold northern environments with a short growing season, where metabolic activities and growth rates are limited by low temperatures and unfavorable soil conditions (Friend & Woodward, 1990; Larcher, 1980; Oleksyn, Reich, Chalupka, & Tjoelker, 1999; Oleksyn, Tjoelker, & Reich, 1992; Reich, Walters, & Ellsworth, 1992; Reich et al., 1996).

The finding of this study that provenances did not differ (*p *>* *.05) significantly in terms of survival is similar to that of Rweyongeza, Yang, et al. (2007), from a study of *P. contorta* and *P. banksiana* provenances. In conifers, survival often shows genetic variation patterns that differ from those seen in growth characteristics (Andalo et al., 2005; Eriksson, Anderson, Eiche, Ifver, & Persson, 1980; Persson, 1994; Schmidtling, 1994; Wei et al., 2004). Once trees grow past a height of two meters, they develop a high capacity to buffer against climate deterioration and low mortality, and are able to cope with diverse climatic stresses during their lifetime (Kullman, 1987; Persson, 1998). However, the conservative growth pattern developed by higher latitude provenances in response to the harsh climatic conditions at their source stands may have resulted in the absence of provenance effect on survival. The adaptation of lower latitude provenances to the mild climatic conditions from which they originate results in their better growth performance from increased carbon assimilation, compared to higher latitude provenances when grown in a common garden but they can be affected by damage from late frosts, pest attacks, and disease that can cause them to incur higher mortality rates than higher latitude provenances (Campbell, 1979; Korner, 2003; Savva et al., 2007; Vitasse, Delzon, Bresson, Michalet, & Kremer, 2009; Zobel & Talbert, 1984). Higher latitude provenances have been adapted to lower winter temperatures and shorter growing seasons of colder climates as well as longer and deeper periods of dormancy they experience at their source sites compared to lower latitude provenances, enabling them to survive adverse conditions. As a result, conservative, slower growth of higher latitude provenances may be a resource allocation strategy directed at survival relative to productivity, developed in response to the climatic conditions at the provenance source stand (Lechowicz, 1984; Leinonen & Hanninen, 2002; Oleksyn, Reich, Zytkowiak, Karolewski, & Tjoelker, 2003; Schmidtling, 1994; Vitasse et al., 2009).

The results show that temperature has a stronger influence on DBH, H, and CW of balsam fir. Precipitation, in combination with the heat accumulation index (moisture index), shows an influence on DBH and H. Temperature and precipitation both had influences on survival. Several studies (Matyas & Yeatman, 1992); Schmidtling, 1994; Parker & Niejenhuis, 1996;  Matyas, 1997; Thomson et al., 2009) have similarly reported that temperature is a better determinant of variation in plant populations, compared to precipitation. The results suggest a strong adaptation of balsam fir provenances to the temperature at their seed sources. The balance between selection and gene flow is influential in local adaptation. Balsam fir, like most conifers, is monecious. However, conifers commonly cross‐pollinate because of the location of female and male reproductive structures in the upper and lower branches of the tree crown, respectively, and the flowering of such structures not coinciding precisely. This is, in addition to wind pollination, characteristic of conifer life history (Barnes, Zak, Denton, & Spurr, 1998; Frank, 1990; Pallardy, 2008). Such circumstances would facilitate gene flow among species populations and could minimize selective pressure that would engender adaptation to local conditions at the provenance sites. Adaptation to local temperature conditions, as the results show, could therefore be an indication of relatively strong directional selection in the species, which may have been facilitated by the spatial predictability of temperature with latitude (Andalo et al., 2005; Arend, Kuster, Günthardt‐Goerg, & Dobbertin, 2011; Oleksyn, Tjoelker, & Reich, 1998).

The annual moisture index based on GDD10 encompasses the growing season, during which tree diameter growth from cambial activity occurs in spring and early summer (Barnes et al., 1998). Although the lower and higher latitude provenances had comparable annual precipitation levels at their seed sources, the higher GDD10 levels at the lower latitude provenance seed sources may have contributed to adaptations in them, which resulted in earlier reactivation of cambial activity than in the higher latitude provenances. Key to cambial reactivation and growth is spring temperature and soil water availability during the growing season (Barnes et al., 1998; Deslauriers, Rossi, Anfodillo, & Saracino, 2008; Gricar, Zupancic, Cufar, & Oven, 2007; Gricar et al., 2006; Kirdyanov, Hughes, Vaganov, Schweingruber, & Silkin, 2003; Oribe, Funada, & Kubo, 2003; Oribe, Funada, Shibagaki, & Kubo, 2001). The response of H to spring moisture index based on GDD10 is indicative of the importance of spring events to height growth. Height growth commences early, often prior to the last frost, and concludes in the early part of the growing season (Baldwin, 1931; Barnes et al., 1998; Cook, 1941; Husch, 1959; Kozlowski, 1955, 1962; Kozlowski & Ward, 1957a,b; Kramer, 1943; Salminen & Jalkanen, 2005; Zimmerman & Brown, 1974). As with annual precipitation, the lower and higher latitude provenances had comparable spring precipitation levels at seed source, but the higher GDD10 values at the lower latitude provenance seed sources may also have engendered adaptations, which resulted in earlier spring budding activity, for which spring warming is an important factor (Barnes et al., 1998). Crown width is an important factor in tree growth, as the crowns of trees are the means by which they intercept and absorb solar radiation, and the location of physiological processes, such as photosynthesis, respiration, and transpiration (Honer, 1971; Grace, 1990; Wang & Jarvis, 1990; Stenberg et al., 1994; Vose et al., 1994; McCrady & Jokela, 1996, 1998; Xiao, Jokela, & White, 2003; Crecente‐Campo et al. 2009). The variation of CW among provenance sources in response to mean minimum annual temperature may be indicative of the restrictive effect of very low winter temperatures on CW (Bechtold, 2003) of the higher latitude provenances of the Prairie Ecozone, compared to the lower latitude provenances of the Atlantic Maritime Ecozone. The relationships of survival with total precipitation, the number of days with precipitation above 10 mm, and temperature below −20°C are in agreement with the findings of Rweyongeza, Dhir, et al. (2007), who reported that survival in white spruce (*Picea* spp.) provenances responded best to precipitation over the course of the year, and cool seasonal temperatures.

These results suggest that the response of balsam fir to climatic variation will likely not be uniform in the species, but differ based on genetic characteristics between populations located in the northern and southern parts of the species’ range. Population differences in response to climatic variation may be evident earlier in growth traits, compared to survival in balsam fir. The findings of this study will facilitate modeling in the species that is reflective of genetic variation in response to climatic conditions, and guide provenance selection for utilization in terms of productivity or resilience as well as breeding programs directed at obtaining species that possibly combine both traits.

## 5 CONCLUSION

This study investigated the effect of climatic variation on morphological traits of balsam fir provenances growing in a common garden in northern New Brunswick. The results showed that lower latitude provenances performed significantly better than higher latitude provenances (*p* < .05), with regard to DBH, H, and CW, indicative of genotypic control of these traits. The lack of a significant difference among provenances (*p* > .05) with regard to survival is reflective of a resource allocation strategy directed at survival relative to productivity arising from genetic adaptations in higher latitude provenances, which, although resulting in slower growth compared to the lower latitude provenances, facilitates a lowering of mortality rates under adverse conditions. Temperature had a stronger relationship with DBH, H, and CW than precipitation. Both climatic variables had some effect on survival. The relationship of temperature with DBH, H, and CW suggests strong directional selection in balsam fir that has engendered adaptation to local conditions in populations of the species along latitudinal gradients. The results suggest that the response of balsam fir to climatic variation will likely differ between populations located in the northern and southern parts of the species range. Population differences in response to climatic variation may be evident earlier in growth traits compared to survival in the species. These findings will facilitate modeling for balsam fir that is reflective of genetic variation in response to climatic conditions, and guide provenance selection for utilization and breeding programs.

## CONFLICT OF INTEREST

None declared.

## AUTHOR CONTRIBUTION

MEA and CPAB conceived the ideas; MEA collected the data from Natural Resources Canada, designed the methods used, analyzed the data, and led the writing of the manuscript; CPAB reviewed the drafts, made amendments to the manuscript, and gave final approval for its publication.
